# Correction: Potential greenhouse gas reductions from Natural Climate Solutions in Oregon, USA

**DOI:** 10.1371/journal.pone.0232651

**Published:** 2020-04-29

**Authors:** The PLOS ONE Staff

## Notice of republication

This article was republished on April 17, 2020, to correct an error in the Data Availability statement. The publisher apologizes for the error. Please download this article again to view the correct version. The originally published, uncorrected article and the republished, corrected articles are provided here for reference.

## Supporting information

S1 File Originally published, uncorrected article. (PDF)

S2 File Republished, corrected article. (PDF)

## References

[1] Graves RA, Haugo RD, Holz A, Nielsen-Pincus M, Jones A, Kellogg B, et al (2020) Potential greenhouse gas reductions from Natural Climate Solutions in Oregon, USA. PLoS ONE 15(4): e0230424 10.1371/journal.pone.0230424 32275725 PMC7147789

