# Supplementary material for: Potential greenhouse gas reductions from Natural Climate Solutions in Oregon, USA
Source: PLoS One. 2020 Apr 10;15(4):e0230424. doi: 10.1371/journal.pone.0230424 (PMC7147789; doi:10.1371/journal.pone.0230424)
Supplement: S1 Fig — Deferred timber harvest was applied to counties (shaded green) where less than 50% of the forests are considered at high risk for wildfire. (DOCX) [file pone.0230424.s001.docx]

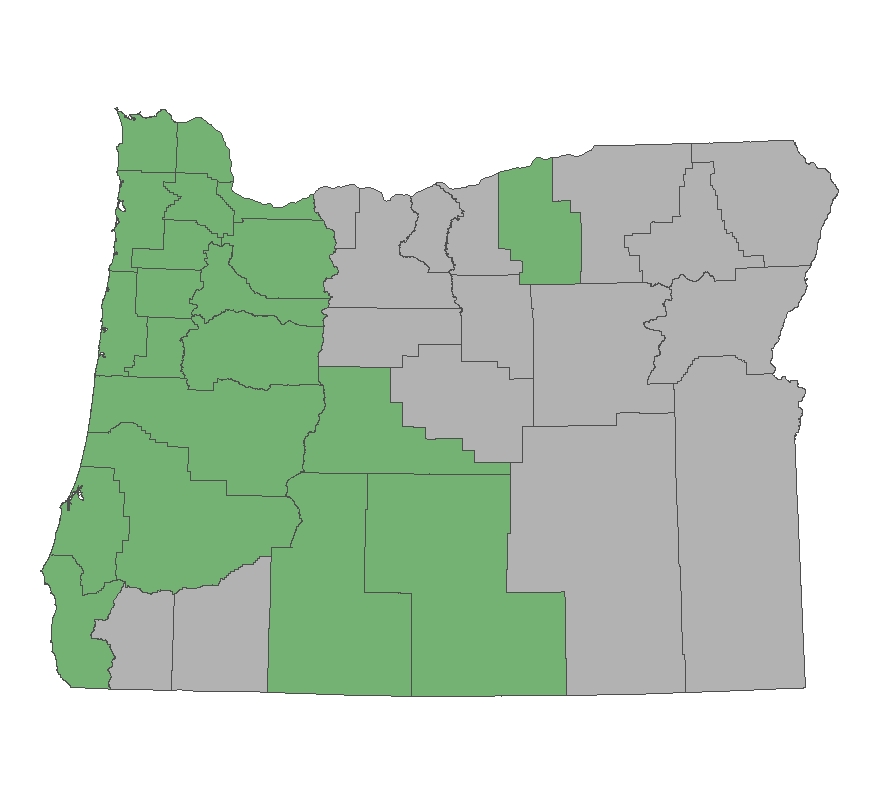


Figure S1. Deferred timber harvest was applied to counties (shaded green) where less than 50% of the forests are considered at high risk for wildfire.
