# Supplementary material for: Potential greenhouse gas reductions from Natural Climate Solutions in Oregon, USA
Source: PLoS One. 2020 Apr 10;15(4):e0230424. doi: 10.1371/journal.pone.0230424 (PMC7147789; doi:10.1371/journal.pone.0230424)
Supplement: S2 Fig — Multiple ownerships provide timber volume (A) and greenhouse gas emission reduction (B) under baseline and NCS implementation scenarios of timber harvest. (DOCX) [file pone.0230424.s002.docx]

**
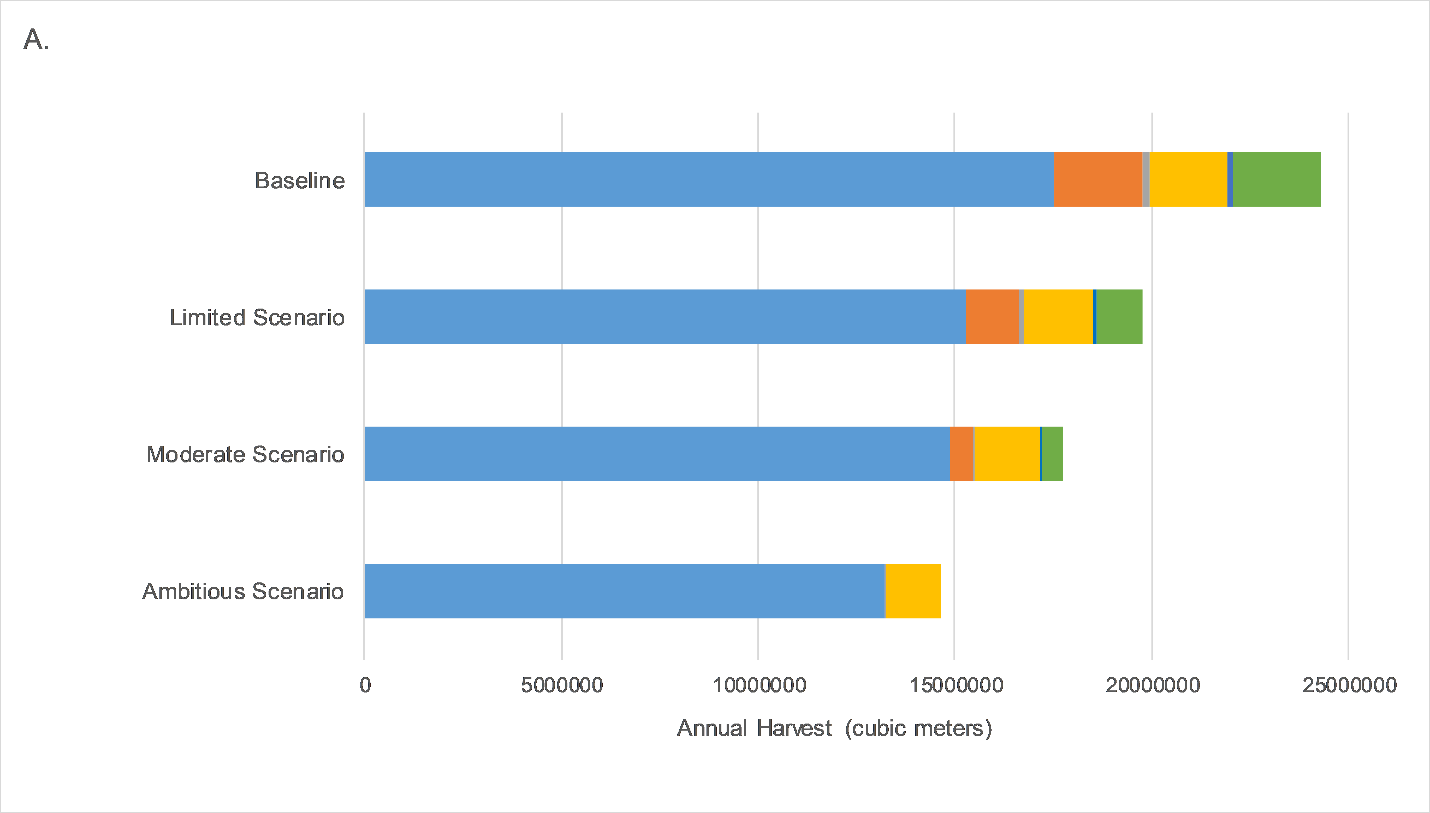

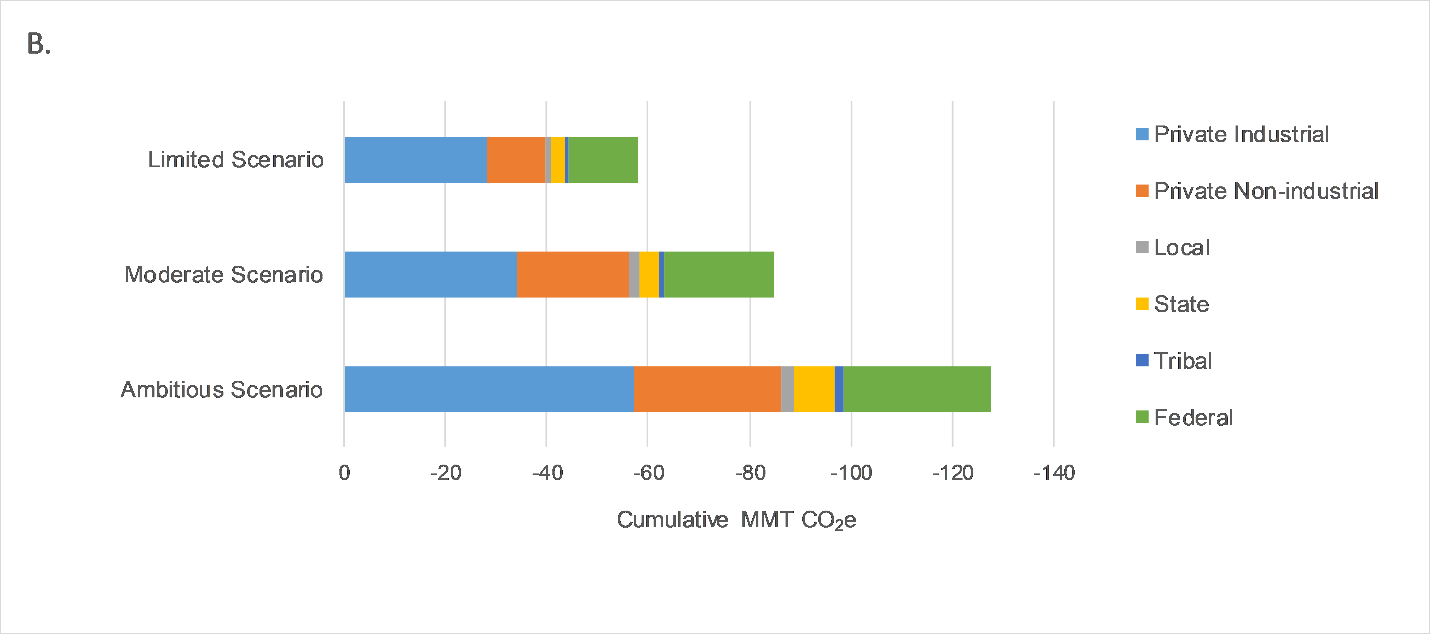
**

**S2 Fig. Multiple ownerships provide timber volume (A) and greenhouse gas emission reduction (B) under baseline and NCS implementation scenarios of timber harvest.**
