# Supplementary material for: Potential greenhouse gas reductions from Natural Climate Solutions in Oregon, USA
Source: PLoS One. 2020 Apr 10;15(4):e0230424. doi: 10.1371/journal.pone.0230424 (PMC7147789; doi:10.1371/journal.pone.0230424)
Supplement: S1 Table — These classifications were used to assign forest productivity rates, simplified to “west” and “east” regions to signify coastal versus interior sensu (1), for most forest-based NCS activities. (DOCX) [file pone.0230424.s003.docx]

**S1 Table. County designation across the interior to coastal productivity gradient.** These classifications were used to assign forest productivity rates, simplified to “west” and “east” regions to signify coastal versus interior *sensu* (1), for most forest-based NCS activities.

| **County** | **Region** |
| --- | --- |
| Baker | East |
| Benton | West |
| Clackamas | West |
| Clatsop | West |
| Columbia | West |
| Coos | West |
| Crook | East |
| Curry | West |
| Deschutes | East |
| Douglas | West |
| Gilliam | East |
| Grant | East |
| Harney | East |
| Hood River | West |
| Jackson | West |
| Jefferson | East |
| Josephine | West |
| Klamath | East |
| Lake | East |
| Lane | West |
| Lincoln | West |
| Linn | West |
| Malheur | East |
| Marion | West |
| Morrow | East |
| Multnomah | West |
| Polk | West |
| Sherman | East |
| Tillamook | West |
| Umatilla | East |
| Union | East |
| Wallowa | East |
| Wasco | East |
| Washington | West |
| Wheeler | East |
| Yamhill | West |
