# Supplementary material for: Potential greenhouse gas reductions from Natural Climate Solutions in Oregon, USA
Source: PLoS One. 2020 Apr 10;15(4):e0230424. doi: 10.1371/journal.pone.0230424 (PMC7147789; doi:10.1371/journal.pone.0230424)
Supplement: S2 Table — Data are summarized from Oregon Department of Forestry harvest data (2000–2017) for the following counties: Benton, Clackamas, Clatsop, Columbia, Coos, Curry, Deschutes, Douglas, Klamath, Lane, Lake, Lincoln, Linn, Marion, Morrow, Multnomah, Polk, Tillamook, Washington, Yamhill. (DOCX) [file pone.0230424.s004.docx]

**S2 Table. Average annual timber harvest in cubic meters (baseline) and percent deferment under each scenario by ownership.** Data are summarized from Oregon Department of Forestry harvest data (2000 – 2017) for the following counties: Benton, Clackamas, Clatsop, Columbia, Coos, Curry, Deschutes, Douglas, Klamath, Lane, Lake, Lincoln, Linn, Marion, Morrow, Multnomah, Polk, Tillamook, Washington, Yamhill.

|  |  |  |  | **% timber harvest deferment (per year)** | | |
| --- | --- | --- | --- | --- | --- | --- |
| **Ownership** | **Region** | **Average annual harvest, 2000 - 2017 (m3)** | **Historical variation** | **Limited Scenario** | **Moderate Scenario** | **Ambitious Scenario** |
| Private industrial | East | 796,965 | 49% | Same as historical variation | Linear decline over 10 years to 15%; 15% reduction 2030 - 2050 | Linear decline over 10 years to 100% |
|  | West | 16,707,557 | 11% |  |  | Linear decline over 10 years to 21%; 21% reduction 2030 - 2050 |
| Private non-industrial | East | 64,462 | 65% | Same as historical variation | Linear decline over 10 years to 75%; 75% from 2030 - 2050 | Linear decline over 10 years to 100% |
|  | West | 2,193,819 | 39% |  |  |  |
| Local & county | East | 1,284 | 100% | Same as historical variation | Linear decline over 10 years to 75%; 75% from 2030 - 2050 | Linear decline over 10 years to 100% |
|  | West | 194,185 | 32% |  |  | Linear decline over 10 years to 100% |
| State | East | 69,087 | 39% | Same as historical variation | Linear decline over 10 years to 15%; 15% reduction 2030 - 2050 | Linear decline over 10 years to 100% |
|  | West | 1,902,800 | 10% |  |  | Linear decline over 10 years to 32%; 32% reduction from 2030 - 2050 |
| Tribal | East | 0 | 0 | Same as historical variation | Linear decline over 10 years to 75%; 75% from 2030 - 2050 | Linear decline over 10 years to 100% |
|  | West | 112,206 | 43% |  |  |  |
| Federal | East | 457,978 | 46% | Same as historical variation | Linear decline over 10 years to 75%; 75% from 2030 - 2050 | Linear decline over 10 years to 100% |
|  | West | 1,781,916 | 48% |  |  |  |
