# Supplementary material for: Potential greenhouse gas reductions from Natural Climate Solutions in Oregon, USA
Source: PLoS One. 2020 Apr 10;15(4):e0230424. doi: 10.1371/journal.pone.0230424 (PMC7147789; doi:10.1371/journal.pone.0230424)
Supplement: S3 Table — Upper and lower bounds of 90% confidence interval are shown in parentheses. (DOCX) [file pone.0230424.s005.docx]

Table S3. Estimated annual reductions in MMTCO_2_e for each NCS activity under three different implementation scenarios in years 2035 and 2050. Upper and lower bounds of 90% confidence interval are shown in parentheses.

| NCS Activity | | Scenario/Year | | | | | |
| --- | --- | --- | --- | --- | --- | --- | --- |
|  |  | Limited | | Moderate | | Ambitious | |
|  |  | 2035 | 2050 | 2035 | 2050 | 2035 | 2050 |
| Avoided Conversion | Forest avoided conversion | -0.05  (-0.04, -0.05) | -0.05  (-0.04, -0.06) | -0.24  (-0.20, -0.27) | -0.26  (-0.22, -0.29) | -0.48  (-0.40, -0.55) | -0.52  (-0.44, -0.58) |
|  | Grassland avoided conversion | -0.006  (-0.005, -0.007) | -0.01  (-0.005, -0.007) | -0.04  (-0.03, -0.05) | -0.06  (-0.05, -0.07) | -0.06  (-0.05, -0.07) | -0.06  (-0.05, -0.07) |
|  | Sagebrush-steppe pathways | -0.01  (-0.005, -0.02) | -0.02  (-0.01, -0.04) | -0.07  (-0.02, -0.13) | -0.19  (-0.04, -0.34) | -0.20  (-0.05, -0.34) | -0.35  (-0.08, -0.60) |
| Land  Management | Deferred timber harvest | -2.31  (-2.08, -2.54) | -2.40  (-2.13, -2.66) | -3.36  (-3.04, -3.68) | -3.45  (-3.11, -3.81) | -5.03  (-4.53, -5.52) | -5.20  (-4.64, -5.75) |
|  | Cover Crops | -0.003  (-0.002, -0.005) | -0.003  (-0.002, -0.005) | -0.13  (-0.07, -0.18) | -0.24  (-0.14, -0.33) | -0.54  (-0.33, -0.78) | -1.06  (-0.65, -1.50) |
|  | No-Till | -0.01  (-0.004, -0.016) | -0.01  (-0.004, -0.016) | -0.13  (-0.06, -0.20) | -0.13  (-0.06, -0.20) | -0.09  (-0.04, -0.14) | -0.17  (-0.07, -0.27) |
|  | Nutrient management | -0.07  (-0.04, -0.10) | -0.07  (-0.04, -0.10) | -0.10  (-0.06, -0.13) | -0.10  (-0.06, -0.14) | -0.16  (-0.09, -0.22) | -0.16  (-0.09, -0.22) |
| Restoration | Post-wildfire replanting (Federal Land) | -0.07  (-0.04, -0.10) | -0.15  (-0.12, -0.19) | -0.12  (-0.06, -0.17) | -0.24  (-0.19, -0.31) | -0.22  (-0.09, -0.34) | -0.45  (-0.32, -0.58) |
|  | Riparian Reforestation | -0.14  (-0.13, -0.15) | -0.21  (-0.20, -0.22) | -0.31  (-0.29, -0.33) | -0.83  (-0.78, -0.88) | -1.47  (-1.39, -1.56) | -1.86  (-1.76, -1.95) |
|  | Tidal Wetland restoration | -0.005  (-0.001, -0.008) | -0.01  (-0.004, -0.02) | -0.01  (-0.003, -0.02) | -0.02  (-0.01, -0.03) | -0.01  (-0.003, -0.02) | -0.03  (-0.01, -0.05) |
| Overall annual reductions | | -2.67  (-2.34, -3.01) | -2.92  (-2.54, -3.31) | -4.45  (-3.75, -5.16) | -5.45  (-4.55,-6.38) | -8.15  (-6.79,-9.50) | -9.74  (-7.91, -11.5) |
