# Supplementary material for: Potential greenhouse gas reductions from Natural Climate Solutions in Oregon, USA
Source: PLoS One. 2020 Apr 10;15(4):e0230424. doi: 10.1371/journal.pone.0230424 (PMC7147789; doi:10.1371/journal.pone.0230424)
Supplement: Attachment — Submitted filename: Response to Reviewers.docx [file pone.0230424.s006.docx]

Response to Reviewers

Detailed Response to Reviewer Comments

*Authors’ response to reviewer comments are in blue and italics.*

Reviewer #1: Graves et al., presented a well written and important manuscript describing the carbon mitigation potential of variaous Natural Climate Solutions (NCS). I believe this manuscript will be of interest to many scientists and policy makers examining mechanisms of carbon sequstration and mitigation. This is especially important as Oregon makes moves to regulate carbon emissions through cap and trade or other mechanisms.

*Thank you for your positive comments on the utility of our research.*

I have several suggestions to improve the manuscript, but my main criticism is that the coparisons between some of the NCSs are maybe not appropriate. The basis of some of hte NCSs are the entire landbase - timber harvesting for example is based on all land across all ownerships. Whereas, riparian restoration is only based on the current rate of restorration. The authors point out that their calculated rate of riparian restoration may be an underestimate because they onyl use the rate of restortion reported by a couple agencies. However, this rate is probably huge underestimate given the length of streams and rivers that pass thorugh agricultural and urban landscapes in need of restoration (development or harvesting up to stream edge). At the very least, I would like to see the authors make an attempt at determining the amount of C that could be sequestered in riparian zone if they were all restored - you could use OFPA to determine the width of riparian zones by stream type (interesting that once managed forest land is converted to urban or ag the riparian zones can be almost non-exisent, I digress). As it stands, the basis for each of these NCSs is different and so tough to compare.

*We respectfully disagree with the Reviewer’s assertion that comparisons across NCS activities are not appropriate. In all cases, increased NCS implementation in the Limited, Moderate, and Ambitious Scenarios is based on a change from the baseline rate of an activity. In some cases, an activity occurs across multiple ownerships and is reported based on those ownerships (i.e., timber harvest). In other cases, reported baseline rates of an activity are not tied to ownership categories (i.e., riparian reforestation). We used a similar approach for all of the NCS activities, regardless of whether they applied to all ownerships or only a specific portion of the land base. Furthermore, the comparisons presented among NCS in our study are similar to published comparisons of GHG reduction activities due to NCS (Cameron et al. 2017, Griscom et al. 2017, Fargione et al. 2018). As with these published estimates, we do not claim to provide a complete inventory and assessment of all possible pathways but rather to highlight and start a conversation around the role of NCS in climate change mitigation.*

*We have addressed the Reviewer’s concern with respect to the underestimate of riparian area restoration. We maintain our current methodology for establishing the baseline annual rate of restoration [see lines 380-387] as the best first approximation based on the data available. No current data products, spatial or otherwise, exist which accurately identify statewide riparian restoration efforts. We rely on reporting to two of the largest riparian restoration funding agencies within the State (Oregon Watershed Enhancement Board and the Natural Resource Conservation Service). Based on these data, we estimate that the current rate of riparian reforestation (i.e., restoration plantings) is 2395 ha per year, with an average of 1712 ha per year replanted east of the Cascades and 683 ha per year replanted west of the Cascades.*

*To address the Reviewer’s concern, we have improved on the upper bounds of riparian reforestation potential used in the Ambitious Scenario estimate of carbon sequestration potential. While detailed maps of current riparian condition exist for individual sections of selected watersheds throughout Oregon, a comprehensive statewide assessment of riparian condition has not been conducted for the State of Oregon. The method offered by Reviewer #1 using the Oregon Forest Practices Act (OFPA) stream designations is not sufficient for estimating restorable riparian areas for two main reasons. First, the OFPA buffer requirements vary depending on stream characteristics from 20 to 100 m and are not easily translated to mapped buffers at the state level. Second, while buffering all the streams in the state may provide an estimate of riparian habitat, it still does not adequately describe the current condition of those riparian areas. For example, an oversimplified estimate of statewide riparian areas using 100-m buffers on either side of mapped streams would result in a total area of riparian habitat in Oregon of 36,927 km^2^ but would not take into consideration stream order/size or condition and is likely to overestimate of potential restoration opportunity (Gregory 2000).*

*Instead, we estimate the maximum extent of riparian reforestation opportunity by combining published floodplain maps (Wing et al. 2018) with recent mapped tree canopy cover data (USFS 2019) and environmental site potential (LANDFIRE 2014). We considered areas to have riparian reforestation potential if they (1) occurred within a 100-year floodplain, (2) had less than 40% canopy cover, and (3) had ecological site potential that included forest, woodland, or was undetermined based on biophysical setting. Urban areas are outside the scope of this study and so we did not include areas mapped as high or moderate density development (NLCD 2011) in our estimate of riparian reforestation potential. This resulted in 202,415 ha (2024 km^2^) of potential reforestation across Oregon, the majority of which is located on the west side of the state. We have modified our Ambitious Scenario to reach a cumulative riparian reforestation of 202,415 ha by 2050 [Table 3]. We also included the maximum threshold in all three scenarios, better reflecting the upper limit of riparian reforestation opportunity in the eastern portion of the state. These changes led to increased C sequestration potential from riparian reforestation as compared to our previous estimate in both the Moderate and Ambitious scenarios. Specifically, by 2050, the cumulative C sequestration potential from riparian reforestation increased by 11% or 3.8 MMT CO_2_e in the Ambitious Scenario and by 86% or 5.1 MMT CO_2_e in the Moderate Scenario.*

Specific comments.
Line 112: The authors should consider creating a conceptual diagram or figure that describes their methods.

*Thank you for the suggestion. Rather than create an additional figure or conceptual diagram, we have elected to streamline and simplify the “General Analytical Framework” section and hope that it helps to illustrate the cross-cutting methods.*

Table 1: Consider adding citations to this table

*We draw the definitions of NCS pathways from Griscom et al. (2017), Cameron et al. (2017), and Fargione et al. (2018). We have added these citations to the table caption.*

Line 175-177: Was this arbitrarily chosen? No problem if so, but if guided by some research it needs a citation.

*We have added citations and have revised our estimate of the proportion of carbon sequestration lost due to the conversion of forests to urban and rural land uses [lines 164-166]. Woodbury and colleagues (2007) assessed carbon flux across the United States and estimate that urban/suburban forests sequester carbon at a ratio of 16/100 compared to forests. Thus, we revised our estimates of forgone C sequestration in forests converted to urban land use to be 84% of the current sequestration. For rural land uses, we assume that 50% of the forest cover is retained, loosely following the NLCD low-intensity development definition (<49% impervious cover).*

Reviewer #2: Potential greenhouse gas reductions from Natural Climate Solutions in Oregon, USA
Graves, Haugo, Holz, Nielsen-Pincus, Joens, Kellogg, MacDonald, Popper

Overview
This study sought to determine the relative importance of different natural climate solutions (NCS) on their ability to reduce greenhouse gas emissions. The study was comprehensive, covering many different options for NCS, and the results were interesting, showing that delayed timber harvest was the most important contributor to potential GHG reductions. The paper was well-written, figures were generally clear and the results had state-level policy implications, which was good to see. My main concern was the lack of information in the discussion about the potential importance of different landowners in delaying timber harvest to reduce GHG emissions. If it was mostly private industrial, it would be a lot less practical to implement than say state lands.

*Thank you for your positive review of our manuscript. We appreciate your suggestions and address the specific comments below. With respect to the delayed timber harvest pathway, we have followed your suggestion and added results specifically outlining delayed timber harvest by ownership [see lines 489-497] as well as a discussion of the potential implications the different ownerships have for implementation [see lines 560-584].*

Minor editorial comments are listed below.
Abstract
L31 Clarify “avoided conversion”. Do you mean to non-forest and to ag? I don’t think natural and working land(s) is necessary.
L33. “the” global drawdown
L35. Plural? Natural and working lands.

*We have made the changes suggested above [lines 31 – 35].*

L40. Why only on federal lands? Above it makes it sound like you’re looking across all ownerships

*Each NCS activity has a specific available land base – we only consider additional replanting after wildfire on Federal lands given current requirements for replanting on private lands. We have removed the reference to federal lands in the abstract to avoid confusion and have added a sentence to clarify that each NCS was evaluated based on practice-specific assumptions and applicable land base [line 37].*

L43. Is avoided conversion the highest on a per area basis? You might want to say that rather than saying it’s relatively high.

*Avoided conversion of forests does not have the highest estimated per area GHG reduction (MTCO_2_e ha^-1^ yr^-1^). It is less than riparian reforestation and tidal wetland restoration (see Figure 1). We have not made any changes to the wording here.*

L51. It’s true that it’s dependent on coordination across jurisdictions but you didn’t discuss private industrial or non-industrial shifts so it’s not as relevant here. Somewhere here you need to address the scope of your study in terms of ownership so it’s clear.

*We have removed the reference to jurisdictions and have added a sentence to the abstract to clarify the scope of our study in terms of ownership [lines 37 - 39].*

L53. I would just say state level. *We have made the suggested change.*

Introduction
L60. Avoid “the worst effects of climate change” sounds better. *We have made the suggested change.*
L61. Again lands? *We have made the suggested change.*
L67. “The next” 10 to 15 years. *We have made the suggested change.*
L74. How about just “some states”? I’m not sure it matters if they are “influential” are not, in terms of emissions. *We have made the suggested change.*
L78. First-of-its-kind *We have made the suggested change.*
L81. You repeat “natural and working lands” fairly frequently. This might be a place where NCS belongs anyway. *Thank you for pointing out our repetitive wording. We have made the suggested change.*
L87. Comma before including would be helpful. *We have made the suggested change.*
L88. To develop a carbon policy framework seems unnecessary. Or at least makes the sentence too long and confusing. *We have simplified the wording.*
L89. Land management rather than natural and working lands? *We have made the suggested change.*
L90. Comma before promoting. *We have made the suggested change.*
L94. Comma after lead. *We have made the suggested change.*
L109. Omit “given different…”. I think it detracts from the power of the sentence. *We have made the suggested change.*


Methods
Methods are quite long at 19 pages. Maybe you could shorten the introductory paragraph to each type of NCS.

*We have attempted to streamline the methods section but still retain enough detail to adequately describe our process. Specifically, we have shortened the cross-cutting methods [lines 113 – 129], removed the subheadings under each activity, and shortened the introductory paragraphs for each NCS activity, as suggested by the Reviewer.*

L71. Omit “for testing” *We have made the suggested change.*

Table 1. The text on the left is shifted too far to the right (i.e. Avoided Conversion). Switch to single spacing there? *We have made the suggested change.*

Under nutrient management, it might be better to say “avoided emissions by…” *We have made the suggested change.*

Interesting because salvage logging and site prep is common practice after wildfire in OR. I wonder why you omitted it. Maybe you’ll tell me later.
Figure 1. It’s not exactly clear why you separated ages of replanting. Is it because of stand age? We don’t know when the fires will occur. Or are you trying to stagger it? Anyway, it’s not clear why you analyze years differently here, but not with the other treatments.

*The Reviewer is correct that we do not know when fires will occur. We make the assumption that the disturbance pattern of wildfires (i.e., frequency and extent) will not change from the historical pattern. We simulate fire frequency and extent based off historical trends [lines 350 – 355]. Replanting after wildfire is a single activity, but has varying rates of sequestration compared to natural regeneration after wildfire depending on stand age [lines 342 - 349]. Thus, we include the varying sequestration rates within Figure 1.*

L195. I wasn’t clear if you included the emissions from the wildfires.

*We do not include emissions from the wildfires per se. The estimates of lost biomass and carbon sequestration are based on comparisons of intact sagebrush-steppe communities to invasive annual grass dominated communities.*

L210. Did you assume the avoided emission between aboveground biomass was similar between grasses and crops?

*Following Fargione et al. 2018, we assume that annual aboveground biomass loss is similar in grasslands and croplands. In both, aboveground biomass is annually harvest, burned, grazed, or decomposed within a few years and thus does not contribute to avoided emissions.*

L219. Should be carbon-rich *We have made the suggested change, and have moved this sentence to the discussion.*

L219. Add comma after world. *We respectfully disagree, as there are not two independent clauses in this sentence.*

L222. Omit “in-forest” throughout the paper. Or just put forest. *We have made the suggested change.*

L231. Interesting. So this over-estimates the emissions now, but they will occur after the simulation. *The Reviewer is correct. At the end of the 30-years, if harvest levels return to current levels the emissions will return to current levels but forest carbon stocks will be higher [as stated Line 222-226].*

L242. Belowground is one word. *We have changed this throughout the manuscript.*

L275. These figures seem like they belong in the results. *To remain consistent in our organization across NCS activities, we have retained the numbers referenced here (i.e., the per unit mitigation potential). The estimates of per unit mitigation potential from NCS activities are intermediate methods and are not considered main results in our study.*

L286. You say it could be used on most of the ag acres, but you haven’t gotten to the point where you tell us how much its being used already. How about Cover crops can be used in Oregon…”? *We have made the suggested change to this sentence, and also have moved this sentence to the discussion.*

L307. You don’t tell us why no-till is important, like you did for cover crops. And you put national figures here but not in the cover crops. It would be good to be consistent in the text across treatments. *We have changed the organization of our Methods section and moved this text (along with other introductory text for NCS activities) to the introduction or discussions sections.*

L364. I don’t understand why you limited it to fed lands when everyone usually replant. I think you just need to clarify your justification. Are you assuming that OFPA could be revoked on federal but not primate land? What about state forested land?

*As the Reviewer indicates, we assume that lands covered by the Oregon Forest Practices Act (i.e., private and state lands) generally are salvage harvested after wildfire, thus triggering the requirement to replant. Therefore, we assume that there is little additional C sequestration opportunity above the business-as-usual management practice. Conversely, current practice on federal lands does not include such high rates of replanting after wildfire [see our estimate of replanting rates on Federal land, Table 2] and thus represents an opportunity for increased activity above the business-as-usual management practice.*

Results
Figure 1. What about color coding it like you did Figure 3? *We have made the suggested change.*

Figure 2. You focus on 2035, but it’s not marked on your graph. Maybe add a vertical dashed gray line? *We have added 2035 to the x-axis to make this graph easier to read.*

L510. Is the median range you present across all 3 scenarios? I feel like I wanted one value with a SE/SD? Or 3 medians, one for each scenario. *We have modified our results to provide the median along with upper and lower bounds for each scenario.*

Figure 5 resolution is poor. *We have improved the resolution of this figure.*

L550. I’m not sure how it could be as high as 67%. Please clarify. *By 2050, if the State is successful in meeting its current fossil fuel mitigation goals across other sectors (e.g., transportation, energy, housing) then annual GHG emissions will be close to 14 MMTCO_2_e. Our Ambitious Scenario suggests that increased NCS implementation could mitigate an additional 9.74 MMTCO_2_e per year, which is 69% of the remaining GHG emissions in 2050.
We have updated this section for clarity and also to include new results.*

Discussion
L563. I’m not sure I would consider a max of 20% as substantial. *We have changed the wording.*

L596. I was curious to know which landowners provided the most emissions reductions since it was the most important factor. Could you add this analysis to the paper? Also consider adding a paragraph about landowners and their expected flexibility to altering their management practices to defer harvest.

*We have added two figures, in addition to the pre-existing S2 Table, which describe the relative contribution of different ownerships to both the baseline timber harvest as well as the cumulative GHG reductions from deferred harvest in the NCS Scenarios [S2 Fig A & B] and describe this breakdown in our results [lines 486 - 494]. Finally, we have added more of a discussion regarding the different landowners and their potential flexibility/ability to alter their management practices [lines 560 – 584].*

Conclusion
L3667. But changing would include limiting or increasing. Might want to clarify or remove if not necessary. *We changed the wording here.*
