# Supplementary material for: Potential greenhouse gas reductions from Natural Climate Solutions in Oregon, USA
Source: PLoS One. 2020 Apr 10;15(4):e0230424. doi: 10.1371/journal.pone.0230424 (PMC7147789; doi:10.1371/journal.pone.0230424)
Supplement: Attachment — Submitted filename: Gravesetal_PLOS_Review Comments to the Author_v2.docx [file pone.0230424.s007.docx]

Response to Reviewer Comments

*Authors’ response to reviewer comments are in blue and italics.*

Review Comments to the Author

Reviewer #2: The authors have addressed all my comments to my satisfaction. Overall, the manuscript looks good and is publishable. My only concern was about data availability. The authors state that all the data is available in the paper and supplemental, but I couldn't find any numbers etc that would make the work reproducible. Though maybe I missed it.

*Thank you for your comments. In order to make the data availability more transparent and the work reproducible, we will archive the R code used in our simulations as well as the input files on GitHub before publication of the study.*

Reviewer #3: This is an important study summarizing the NCS-effects of conservation, management, and restoration on the land base in Oregon. A strength of the paper is the consideration of various alternative management strategies for many types of land uses.

*Thank you for your positive comments.*

While it may not be specifically an NCS, one emerging use of timber that has a large (potential) GHG reduction effect is the substitution of cross-laminated mass timber for concrete and steel. Concrete and steel create large CO2 emissions. Fain et al. (2018) is not cited. They found that substitution is a key variable when assessing carbon benefits over time. I do not know if such substitution effects fall into the scope of NCS, but regardless it appears to be significant...Fain et al claim for over 100 years, substituting wood for concrete and steel has more carbon benefits than lengthening rotations to 120 years. Similarly, wood pellets substituting for natural gas reduce leakage from the wood products pipeline. Such wood products may decrease the NCS carbon benefit but could have non-negligible contribution to fossil-fuel reduction shown in Fig. 5.

Fain et al, and many other studies, have assessments of different rotation lengths on landscape carbon storage, which would be worth comparing with your results. The numbers are difficult to compare as presented in each study but should be possible to do. The paragraph on lines 548-559 would be a good location to compare the results of this study and those of other studies (for example: Fain, Buotte, Franklin, Harmon, others). A comparison of the quantitative results from these previous studies that consider longer rotations, or partial harvests, should be expected from readers. Such a comparison, and ideally an assessment of which study is more robust, would be very valuable.

*Thank you for the references. We agree that substitution is an important, albeit uncertain, variable in calculating the carbon emissions from timber harvest. We do not include changes in the current wood products pool in our NCS scenarios but have now added a short discussion of the importance of long-lived wood products (such as cross-laminate timber), including references to Fain et al. 2018 [Lines 576 – 588].*

*Of the harvested wood volume considered in our study, 15% is assumed to be unused mill residue or mill residue burned on site. We consider the remaining 85% to be the transformed wood products pool. We further assume that 72% of the transformed wood products in Oregon are long-term storage (i.e., the carbon remains stored in these products for 20 years or more), following Fargione et al. 2018 and Oswalt et al. 2018. While our study considers deferring overall timber harvest as the mechanism to reduce timber harvest emissions, increasing the proportion of transformed wood products in the long-term storage pool would also result in lower timber harvest emissions regardless of the substitution effect.*

Fain, S., Kittler, B., & Chowyuk, A. (2018). Managing Moist Forests of the Pacific Northwest United States for Climate Positive Outcomes. Forests, 9(10), 618. doi: 10.3390/f9100618

Buotte, P. C., Law, B. E., Ripple, W. J., & Berner, L. T. (2019). Carbon sequestration and biodiversity co‐benefits of preserving forests in the western United States. Ecological Applications. doi: 10.1002/eap.2039

*Thank you for the suggested reference to Buotte et al. 2019. This paper was not published at the time of our submission. We now include it, along with others you suggested, in the discussion (Lines 572-573).*

170-174: I do not understand how using the SD of county data is an estimate of uncertainty in C emmissions. There is no expected central tendency in mean C numbers across space. I would expect large regional differences based on topography, rainfall, etc. even within west-side and east-side values. I think that uncertainty should be based upon uncertainty in the original estimates of AGB...which would require examining the methods behind obtaining these estimates. On the other hand, if you can make the argument that county-level estimates for Douglas-fir forest in, say, Clackamas County vs. Josephine County should be similar...then state that is the case.

*We thank the Reviewer for pointing out this shortcoming in our estimate of uncertainty for avoided emissions and lost sequestration potential due to conversion of forests to development. We have updated our methods (see Lines 152-159 and Lines 173 - 177). Specifically, we now take advantage of the ability to report the sampling error within the FIA Evalidator tool. This allowed us to get estimates of the mean carbon in all forest carbon pools (aboveground, belowground, woody debris, litter, and soil organic carbon) as well as the sampling error and the number of plots used for those calculations in each county. We retain our groupings of counties into interior and coastal regions (east side vs. west side) but rather than calculate the standard deviation across the counties, we pool data within each region and calculate a pooled mean and standard deviation for the interior and coastal region. We believe that this provides an adequate estimation of uncertainty for carbon emissions and sequestration in each region.*

193 and 202: random normal distributions? or randomly from a normal distribution? Also, normal distributions have a mean and sd by definition, so do not need to specify that, unless you want to provide the mean and sd values in the text (or at least point to the table that has these data.) Similarly, line 288 and 310...'random?' uniform distribution?

*We have clarified the wording about the distributions used in Monte Carlo simulations throughout our manuscript (including lines 193, 202, 288, 310).*

Minor comments
52: low potential of...

Reviewer #4: I was not one of the previous reviewers for this manuscript. After reading the reviews, the authors’ responses, and the entire paper, I suggest that it be accepted for publication after minor revision. This is a well-done study that will have substantial interest in examining the benefits of NCS in a single state. It is important that global and U.S.-level NCS studies be regionalized to determine the importance of local factors in the potential outcomes. Oregon’s strong land-use laws indeed have a strong impact on what can achieved in NCS because many land-use conversions are already not allowed. All NCS studies are fraught with assumptions based upon imperfect data and uncertainties on what is feasible politically and economically, but the authors make reasonable decisions and clearly state the limitations of their study.

*Thank you for your positive comments and assessment of our study.*

I have a few, mostly minor, suggestions for the authors:

Table 2. MBF should be defined.

Table S2. For average annual harvest, use commas to delineate changes at each 1,000 change and right justify the numbers so that similar digits (i.e., in 10s) line up vertically. Currently, it is very difficult to visually compare the areas.

Line 388. Given the uncertainties in these estimates, round up these rates to the 1's place (i.e., get rid of the numbers to the right of the decimal).

*We have made the suggested changes above (Table 2, Table S2, and Line 388[400 in revised manuscript]).*

Line 652. This is my only substantive criticism. It is good that the Law et al. (2018) article in PNAS is mentioned here, but I think that its findings should be elaborated on more in the discussion. It examines many of the forestry activities in Oregon that are shown to have the highest NCS benefit in this manuscript but with substantially different methods and assumptions. Thus, I believe that it would enhance the discussion substantially to compare the results of the two studies.

*We have added a more thorough comparison of our study with the work of Law et al. (2018) [Lines 567-575]. While a direct comparison of their work with our work is challenging given the different assumptions and accounting metric (i.e., Law and colleagues track the net ecosystem carbon balance whereas we focus solely on avoided emissions or increased sequestration due to changes in land management), our study concurs that limiting timber harvest provides the greatest GHG emission reductions.*
